# Supplementary material for: The Efficacy of Targeted Monoclonal IgA Antibodies Against Pancreatic Ductal Adenocarcinoma
Source: Cells. 2025 Apr 24;14(9):632. doi: 10.3390/cells14090632 (PMC12071589; doi:10.3390/cells14090632)
Supplement: Supplementary file 1 [file cells-14-00632-s001.zip › cells-3569171-supplementary/Supplemental Files/Supplementary material - abbreviations & legenda Suppl Figures.pdf]

Supplementary materials - abbreviations

PDAC	pancreatic ductal adenocarcinoma
TME	tumor microenvironment
mAb(s)	monoclonal antibody (antibodies)
TAA(s)	tumor-associated antigen(s)
EpCAM	epithelial cell adhesion molecule
TROP2 (TACSTD2)	trophoblast cell surface antigen 2
MUC(1)	mucin(-1)
ADCC(s)	antibody-dependent cellular cytotoxicity assay(s)
EGFR	epidermal growth factor receptor
SIRPα	signal regulatory protein alpha
ATCC	American Type Culture Collection
Pen/Step	penicillin-streptomycin
FCS	fetal calf serum
PBS	phosphate buffered saline
RAKU	Regional Academic Cancer Center Utrecht
RBC(s)	red blood cell(s)
PE	phycoerythrin
Cy	cyanine
HER2	human epidermal growth factor receptor 2
FOLR1	folate receptor 1
7-AAD	7-amino-actinomycin D
BV	brilliant violet
AF	Alexa Fluor
51Cr	chromium-51
FITC	fluorescein isothiocyanate
PB	pacific blue
H7	hilite 7
PerCp	peridinin-chlorophyll-protein
PMN(s)	polymorphonuclear leukocyte(s)
WL	whole leukocytes
CPM	counts per minute
SEM	standard error of the mean
TA	tumor-associated

FcαR	Fc alpha receptor

Legenda's supplementary Figures

Supplementary Figure 1 - Tumor-associated antigen (TAA) expression on pancreatic ductal adenocarcinoma (PDAC) cell lines

(**A**) Antibody staining with 10 µg/mL 'primary' target IgA antibody and 'secondary' PE conjugated anti-human IgA antibody (Anti-hIgA-PE). (**B**) Antibody staining 10 µg/mL 'primary' target IgG antibody and 'secondary' APC conjugated anti-mouse IgG antibody (Anti-mIgG-APC). (**C**) Antibody staining for tumor-specific Tn/STn-MUC1 epitopes (CIM301-1 antibody) was measured with flow cytometry. MCF-7 and JurMA were used as positive controls. A representative graph is shown of a least n=3 independent experiments.

Supplementary Figure 2 - Antibody-dependent cellular cytoxicity (ADCC) assays with PDAC cell lines

Whole leukocyte (WL) antibody-dependent cellular cytoxicity (ADCC) assay of (**A**) Capan-2 and (**B**) Panc 10.05 cells comparing IgA and IgG against EpCAM (heING1) and TROP2 (sacituzumab). Polymorphonuclear leukocyte (PMN) ADCC assay of (**C**) AsPC-1 and (**D**) BxPC-3 cell lines with antibodies targeting EpCAM, EGFR (cetuximab) and TROP2 show no efficient lysis. The Mean ± standard error of the mean (SEM) of specific lysis is shown of at least n=3 independent experiments. Independent experiments are performed in technical triplicate. Two-way ANOVA followed by Tukey's post-hoc test was performed. ****$p < 0.0001$.

Supplementary Figure 3 - Gating strategy to determine tumor-associated antigen (TAA) expression of tumor cells

(**A**) Patient tumor sample #14 was analyzed with flow cytometry: Death cells were excluded with To-Pro-3 and CD45 negative events were selected. These cells were gated on population and then single cells from which PE fluorescent intensity (blue-2) was determined as shown by the histograms. The negative control was used to determine the threshold for TAA negativity. The percentage of positive events was determined. (**B**) The percentage of TAA positivity set out in bar graphs of all samples determined with this method. Since the samples still contain other cell types, such as fibroblasts, the epithelial marker EpCAM was used to determine the percentage tumor cells in the sample. The percentage TAAs was compared relative to EpCAM, which was set as 100% (Figure 3A) and the mean fluorescent intensity (MFI) of the TAA positive cells was set out in bar graphs (Figure 3B). If no positive population was present the MFI of the negative peak was taken.

Supplemental Figure 4 – ADCC of tumor sample later diagnosed as cholangiocarcinoma

Polymorhonuclear leukocyte (PMN) antibody-dependent cellular cytotoxicity (ADCC) assay with IgA against EpCAM and pancreatic ductal adenocarcinoma (PDAC) tumor cells isolated via negative MACS selection (CD45, CD31, PDGFRα- and PDGFRβ negative) after red blood cell lysis. The mean

± SEM of specific lysis of a technical triplicate is shown. Two-way ANOVA followed by Tukey's post-hoc test was performed. ***p < 0.001.

Supplementary Figure 5 - Gating strategy to determine cell types in patient tumor

(**A**) Gating strategy of flow cytometry to determine the presence of red blood cells (RBCs, CD235a+), Leukocytes (CD45+) and Tumor cells. (EpCAM+) in tumor samples (**B**) Gating strategy for leukocyte differentiations shows the presence of granulocytes (CD66b+) with the Fc-Alpha receptor (FcαR, CD89+), monocytes (CD14+), T cells (CD3+), B cells (CD20+) and NK cells (CD56+)

Supplementary Figure 6 - Further characterization immune cells subset in patient tumor samples with flow cytometry

(**A**) Monocytes expressing het Fc-alpha receptor (FcαR). (**B**) CD4+ and CD8+ T cells in patient tumors.
