# Supplementary material for: The Efficacy of Targeted Monoclonal IgA Antibodies Against Pancreatic Ductal Adenocarcinoma
Source: Cells. 2025 Apr 24;14(9):632. doi: 10.3390/cells14090632 (PMC12071589; doi:10.3390/cells14090632)
Supplement: Supplementary file 1 [file cells-14-00632-s001.zip › cells-3569171-supplementary/Supplemental Files/Supplementary Table 1.pdf]

Suplementary Table S1 - Reagents and Antibodies

REAGENT or RESOURCE	SOURCE	IDENTIFIER
Antibodies		
Mouse Anti-CD45 Monoclonal Antibody, Phycoerythrin Conjugated, Clone HI30	BD Biosciences	Cat# 555483<br>RRID:AB_395875
PE anti-human CD31	Biolegend	Cat# 303106<br>RRID:AB_314332
PE/Cyanine7 anti-human CD140a (PDGFRα)	Biolegend	Cat# 323507<br>RRID:AB_2565596
PE anti-human CD140b (PDGFRβ)	Biolegend	Cat# 323605<br>RRID:AB_2299493
Anti-PE MicroBeads	Miltenyi Biotec	Cat# 130-048-801<br>RRID:AB_244373
PE anti-human CD326 (Ep-CAM)	Biolegend	Cat# 324206<br>RRID:AB_756080
PE anti-human EGFR	Biolegend	Cat# 352903<br>RRID:AB_10898161
PE anti-human CD340 (erbB2/HER-2)	Biolegend	Cat# 324405<br>RRID:AB_756121
PE anti-human FOLR1 (Folate Binding Protein)	Biolegend	Cat# 908303<br>RRID:AB_2629794
PE anti-human TACSTD2 (TROP2)	Biolegend	Cat# 363803<br>RRID:AB_2572021
PE anti-human CD227 (MUC-1)	Biolegend	Cat# 355603<br>RRID:AB_2561643
PE anti-human CD47	Biolegend	Cat# 323108<br>RRID:AB_756138
IgA3.0 heING1, anti-human EpCAM	CTI Leusen / UMC Utrecht	
IgG1 heING1, anti-human EpCAM	CTI Leusen / UMC Utrecht	
IgA3.0 cetuximab, anti-human EGFR	CTI Leusen / UMC Utrecht	
IgG1 cetuximab, anti-human EGFR	CTI Leusen / UMC Utrecht	
IgA3.0 sacituzumab, anti-human TROP2	CTI Leusen / UMC Utrecht	
IgG1 sacituzumab, anti-human TROP2	CTI Leusen / UMC Utrecht	
IgA3.0 trastuzumab, anti-human HER2	CTI Leusen / UMC Utrecht	
IgA3.0 ch14,18, anti-human GD2	CTI Leusen / UMC Utrecht	
IgA3.0 ch2448, anti-human Annexin	CTI Leusen / UMC Utrecht	
IgA3.0 3C2, anti-human Mesothelin	CTI Leusen / UMC Utrecht	
IgA3.0 41D12, anti-human CD70	CTI Leusen / UMC Utrecht	
IgA3.0 STRO-002, anti-human FOLR1	CTI Leusen / UMC Utrecht	
CD47 blocker, IgG1 LALAPG SIRPα fusion protein	CTI Leusen / UMC Utrecht	

IgG1 anti-mouse MUC1-ED 139H2	Dr. K. Strijbis / UU / NKI / produced in house	Described in https://www.life-science-alliance.org/content/7/6/e202302366 and https://onlinelibrary.wiley.com/doi/abs/10.1002/ijc.2910340210
IgG1 anti-mouse MUC1-ED 214D4	Dr. K. Strijbis / UU / NKI	A kind gift of John Hilkens. Described in https://onlinelibrary.wiley.com/doi/abs/10.1002/ijc.2910340210
IgG1 anti-mouse MUC4-ED 8G7	Dr. K. Strijbis / UU / Santa Cruz Biotechnology	Cat# sc-53945 RRID:AB_784655
IgG1 anti-mouse MUC16-SEA 5B9	Dr. K. Strijbis / UU / produced in house	Hybridoma a kind gift of Ulla Mandel, University of Copenhagen, Denmark, Described in https://pubs.acs.org/doi/10.1021/pr500215g
CIM301-1, IgG1 anti-human Tn/STn MUC1	Dr. W. Germeraad / MUMC	Described in https://pmc.ncbi.nlm.nih.gov/articles/PMC8197514/
Goat F(ab)2 Anti-Human IgA-PE	Southern Biotech	Cat# 2052-09 RRID:AB_2687523
Goat F(ab')2 Anti-Human IgG-PE	Southern Biotech	Cat# 2042-09 RRID:AB_2795662
Goat F(ab')2 Anti-Mouse IgG(H+L), Human ads-APC	Southern Biotech	Cat# 1032-11 RRID:AB_2794324
Purified anti-human CD326 (Ep-CAM)	Biolegend	Cat# 324201 RRID:AB_756075
Purified anti-human EGFR	Biolegend	Cat# 352901 RRID:AB_10916396
Trop2 (EGP-1) Monoclonal Antibody (MR54)	Thermo Fisher	Cat# 14-6024-82 RRID:AB_10853488
Purified anti-human CD47	Biolegend	Cat# 323102 RRID:AB_756132
Goat F(ab')2 Anti-Mouse IgG-FITC	Agilent	Cat# K007811-8, K0078
Brilliant Violet 510(TM) anti-human CD45	Biolegend	Cat# 304036 RRID:AB_2561940
Alexa Fluor(R) 647 anti-human CD66b	Biolegend	Cat# 305110 RRID:AB_2563171
Mouse Anti-CD89 Monoclonal Antibody, Phycoerythrin Conjugated, Clone A59	BD Biosciences	Cat# 555686 RRID:AB_396037
Anti-Human CD14 Monoclonal Antibody, FITC Conjugated, Clone TÜK4	Miltenyi Biotec	Cat# 130-080-701 RRID:AB_244303
Mouse Anti-CD3 Monoclonal Antibody, Pacific Blue Conjugated, Clone UCHT1	BD Biosciences	Cat# 558117 RRID:AB_397038
Mouse Anti-Human CD20 Monoclonal Antibody, APC-H7 Conjugated, Clone 2H7	BD Biosciences	Cat# 560734 RRID:AB_1727449

CD56 PE-Cy7, clone NCAM 16.2	BD Biosciences	Cat#335826<br>RRID:AB_2857328
PE anti-human LOX-1	Biolegend	Cat# 358604<br>RRID:AB_2562181
Mouse Anti-Human CD14 Monoclonal Antibody, APC-H7 Conjugated, clone MϕP9	BD Biosciences	Cat# 560180<br>RRID:AB_1645464
Pacific Blue(TM) anti-human HLA-DR	Biolegend	Cat# 307624<br>RRID:AB_493665
FITC anti-human CD68	Biolegend	Cat# 333806<br>RRID:AB_1089054
APC anti-human CD4	Biolegend	Cat# 300514<br>RRID:AB_314082
PE anti-human CD8a	Biolegend	Cat# 301008<br>RRID:AB_314126
PerCP-Cy5.5 anti-human CD25	Sony Biotechnology	Cat# 2113130
Anti-Human Foxp3 Monoclonal Antibody, Alexa Fluor 488 Conjugated, Clone PCH101	Thermo Fisher	Cat# 53-4776-73<br>RRID:AB_469911
7-AAD Staining Solution	BD Biosciences	Cat# 559925<br>RRID:AB_2869266
TO-PRO-3 iodide (642/661)	Thermo Fisher	Cat# T3605, Lot# 2069619
Chemicals, peptides, and recombinant proteins		
RPMI 1640 with Hepes and GlutaMAX	Thermo Fisher, Gibco	Cat# 72400-021
IMDM	Thermo Fisher, Gibco	Cat# 12539089
McCoy's 5A (Modified) Medium	Thermo Fisher, Gibco	Cat# 15410604
Penicillin/Streptomycin	Thermo Fisher, Gibco	Cat# 11548876
Fetal calf serum, heat inactivated	Sigma-Aldrich	Cat# F7524
Phosphate buffered saline (PBS), pH7.4	Produced in house	
Trypsin-EDTA 0.5% 10x	Thermo Fisher, Gibco	Cat# 15400-054
MACS Tissue Storage Solution	Miltenyi Biotec	Cat# 130-100-008
RBC lysis buffer 10x	Biolegend	Cat# 420302
BSA, 0.5%	Roche diagnostic	Cat# 10735094001
NaN3 sodium azide, 0.1%	Thistle scientific	Cat# SBL-40-2010-01
Chromium-51, 100µCi	PerkinElmer	
Ficoll Paque Plus	GE Healthcare	Cat# 17-1440-03
Triton-X-100, 5%	Sigma-Aldrich	Cat# X100
Commercial assays		
MycoAlert mycoplasma detection kit	Lonza	Cat# LT07-318
Human Tumor Dissociation Kit	Miltenyi Biotec	Cat# 130-095-929
QIFIKIT (Quantitative Analysis Kit)	Agilent	Cat# K007811-8
Experimental models: Cell lines		
AsPC-1	ATCC	Cat# CRL-1682<br>RRID:CVCL_0152
BxPC-3	ATCC	Cat# CRL-1687<br>RRID:CVCL_0186
Capan-2	ATCC	Cat# HTB-80<br>RRID:CVCL_0026
CFPAC-1	ATCC	Cat# CRL-1918<br>RRID:CVCL_1119
Panc 10.05	ATCC	Cat# CRL-2547<br>RRID:CVCL_1639
MCF-7	ATCC	Cat# HTB-22<br>RRID:CVCL_0031

JurMA (J.RT3-T3.5)	ATCC	Cat# TIB-153<br>RRID:CVCL_1316
**Software**		
FlowJo v10	TreeStar	https://www.flowjo.com/solutions/flowjo/
GraphPad Prism 10.1.2	GraphPad Software Incorporated	https://www.graphpad.com/
**Other**		
GentleMACS Octo Dissociater with heaters	Miltenyi Biotec	Cat# 130-096-427
QuadroMACS Starting Kit (LD)	Miltenyi Biotec	Cat# 130-092-857
MACS MultiStand	Miltenyi Biotec	Cat# 130-042-303
LD columns	Miltenyi Biotec	Cat# 130-042-901
FACS Canto II Flow Cytometer	BS Biosciences	https://www.bdbiosciences.com/en-se/products/instruments/flow-cytometers/clinical-cell-analyzers/facscanto
LumaPlate-96	Revvity	Cat# 6006633
Beta-gamma counter	PerkinElmer	
