# Supplementary figures and images for: The Efficacy of Targeted Monoclonal IgA Antibodies Against Pancreatic Ductal Adenocarcinoma
Source: Cells. 2025 Apr 24;14(9):632. doi: 10.3390/cells14090632 (PMC12071589; doi:10.3390/cells14090632)

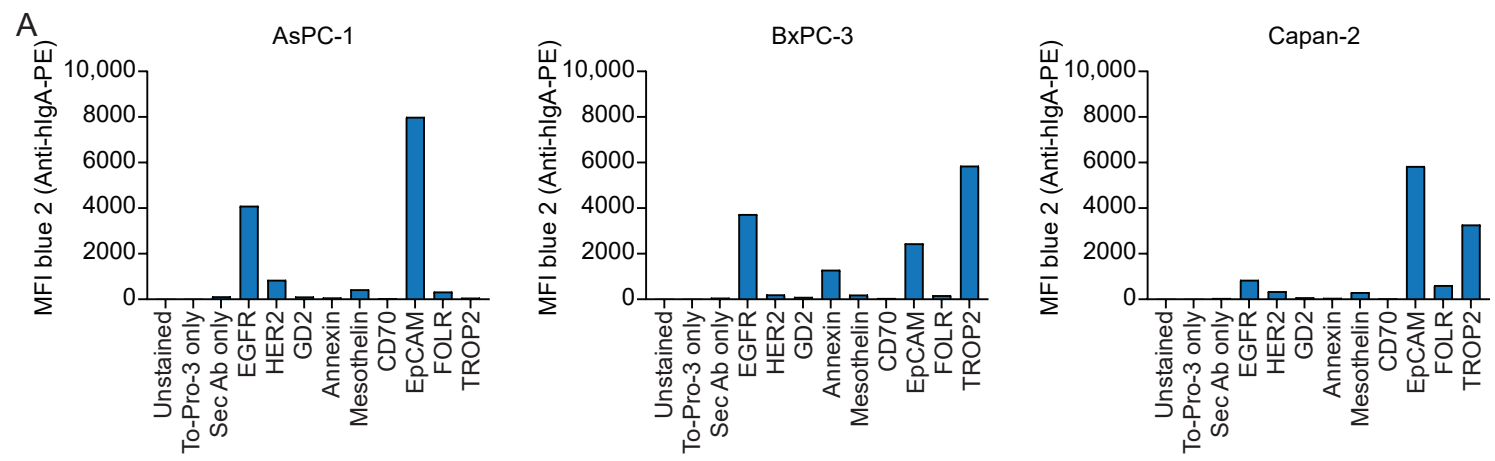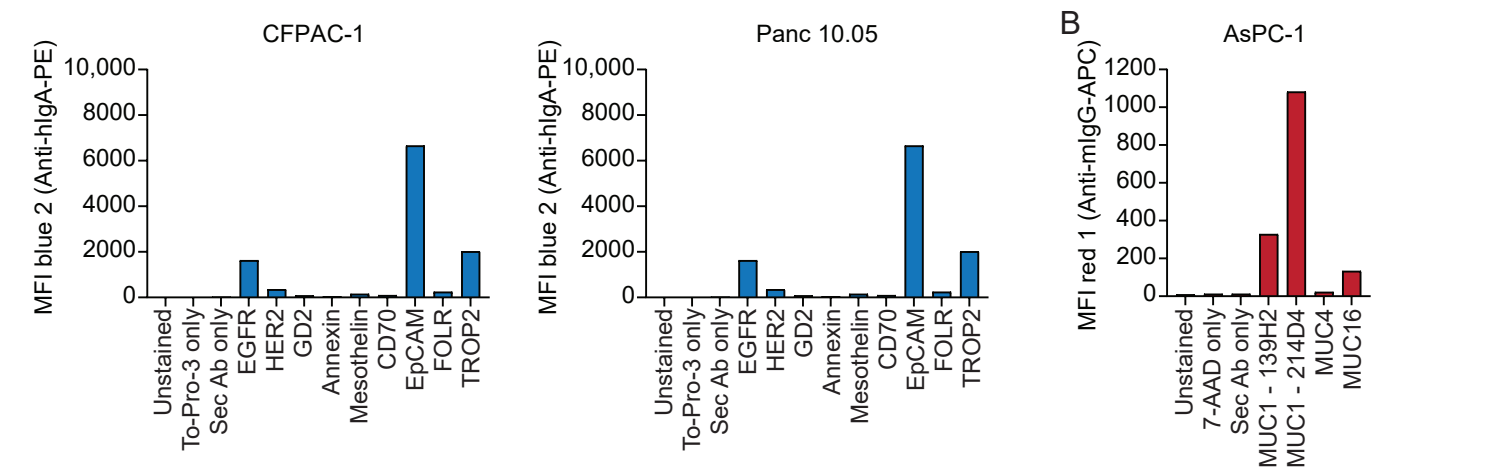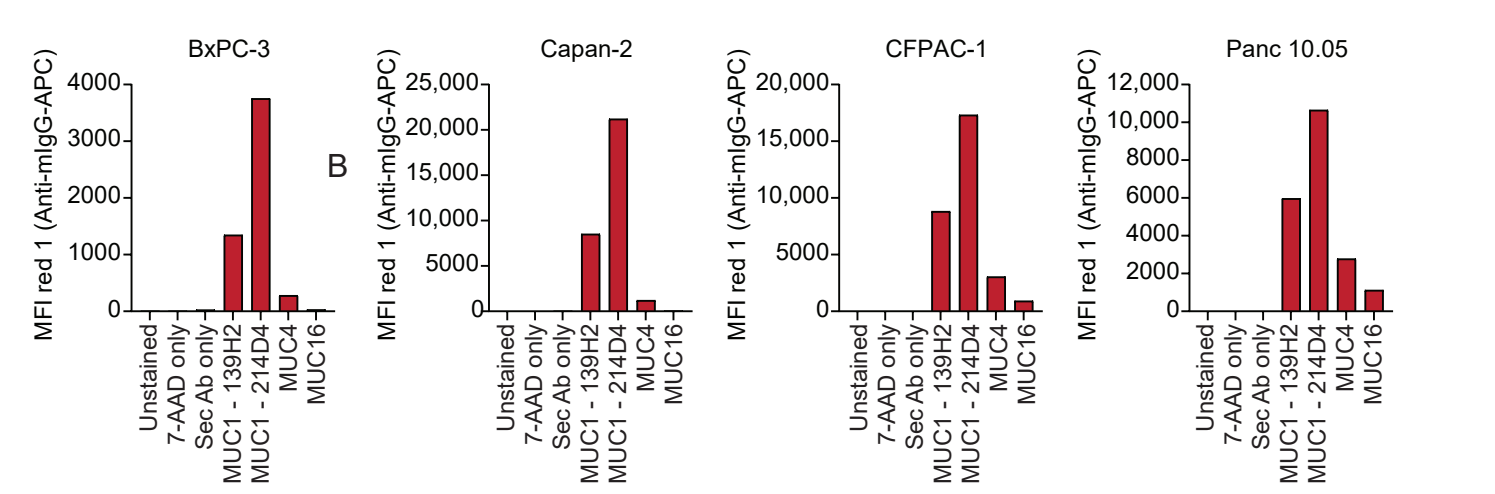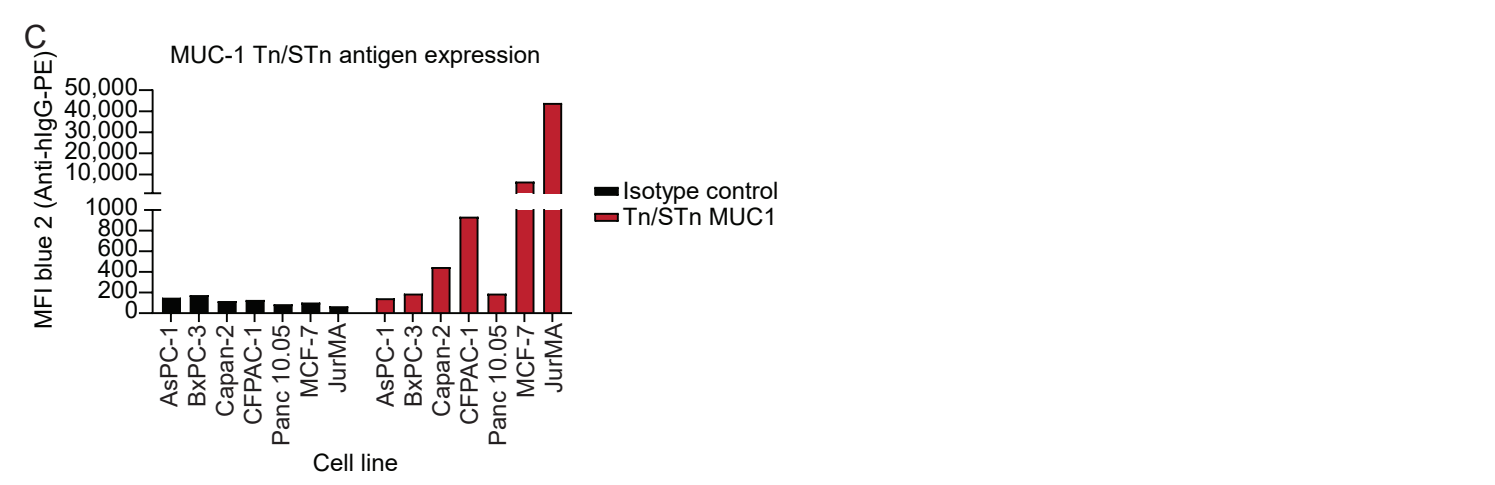

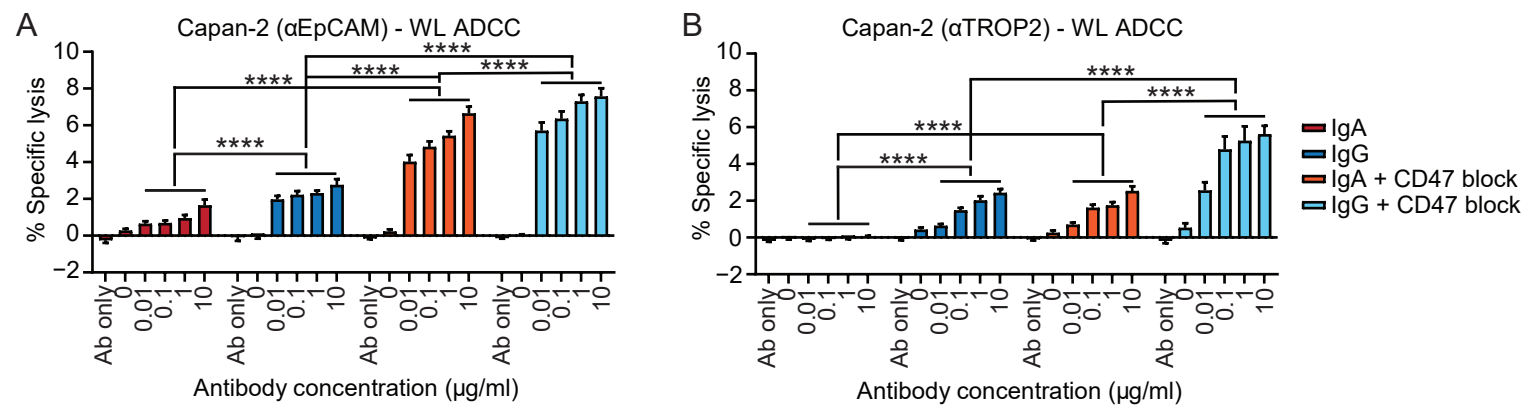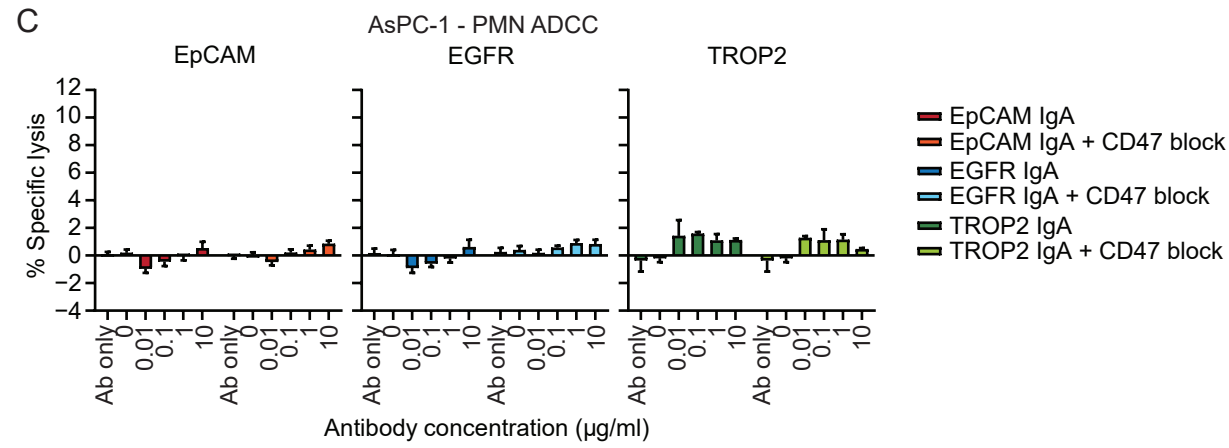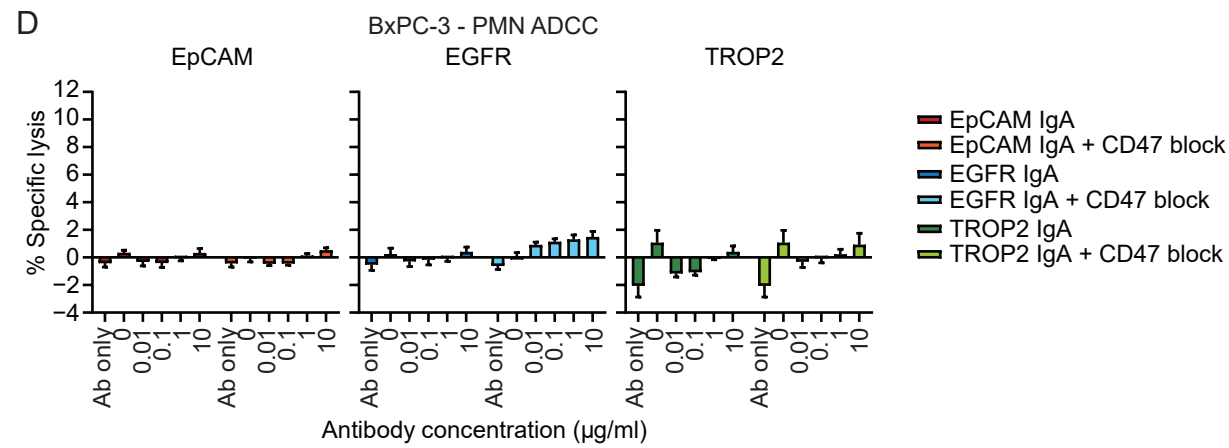

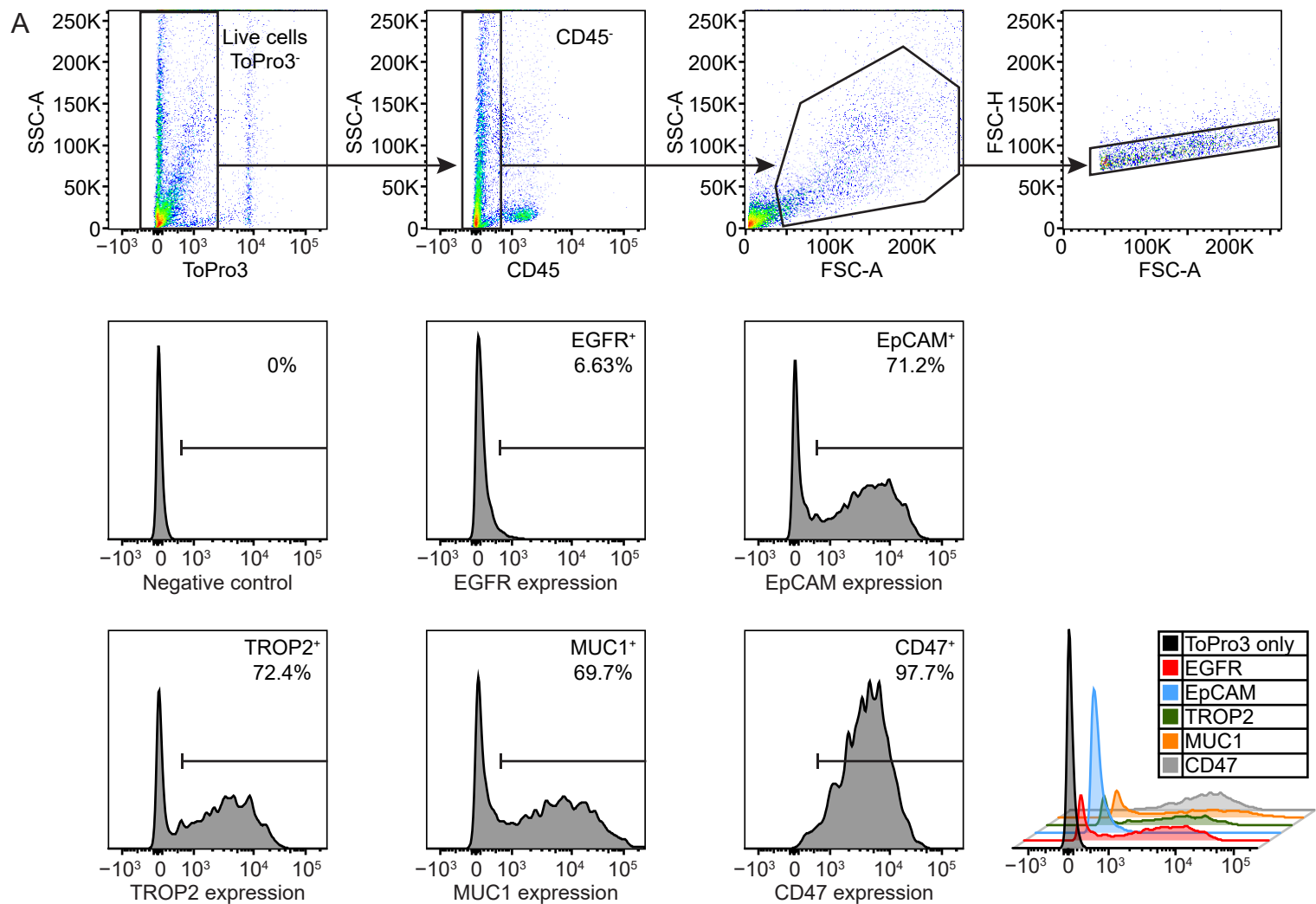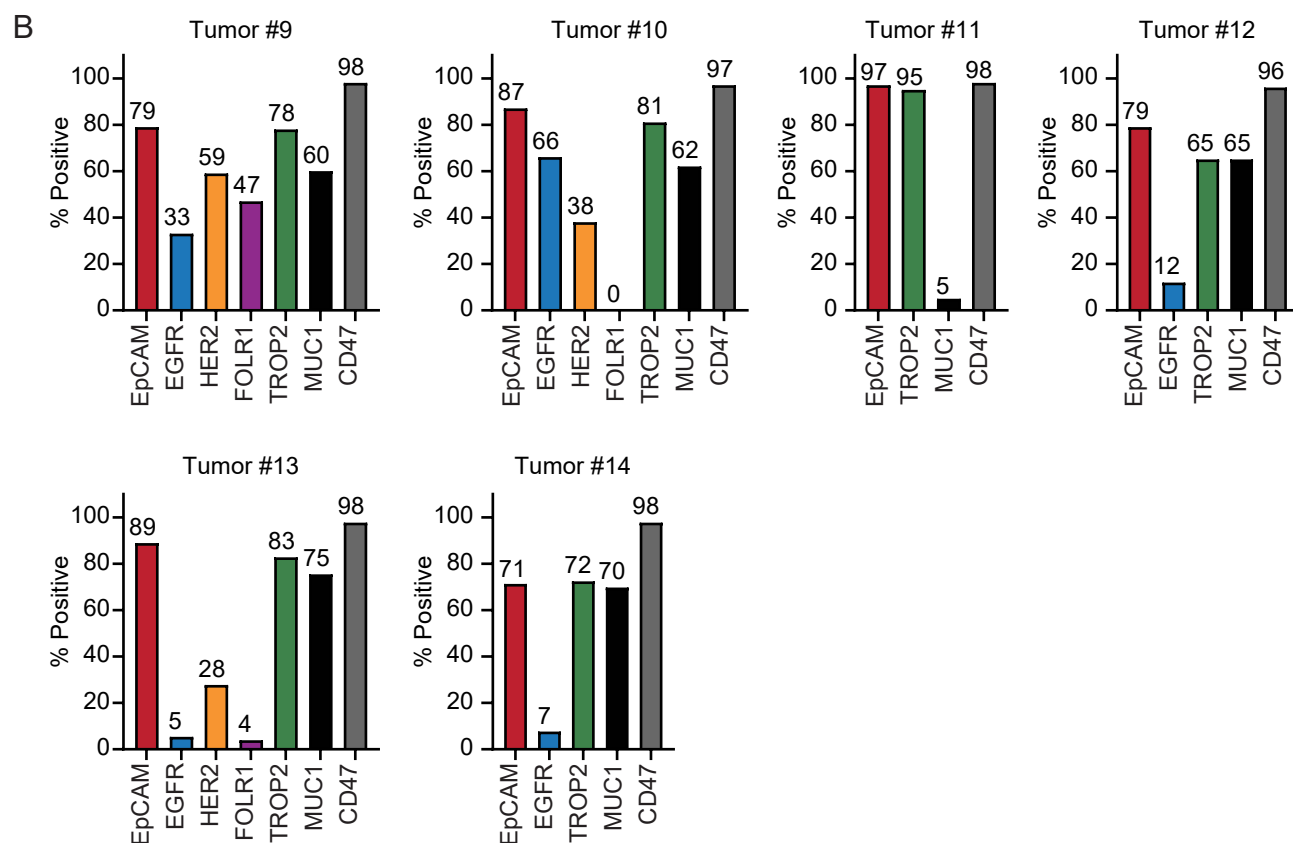

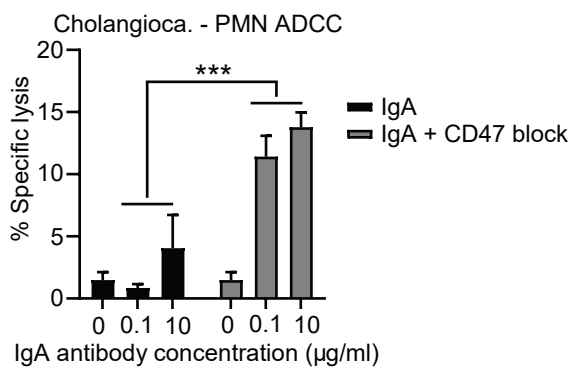



A

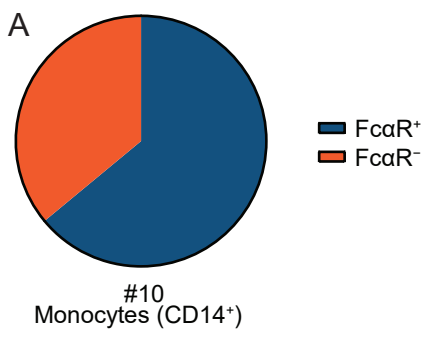

B

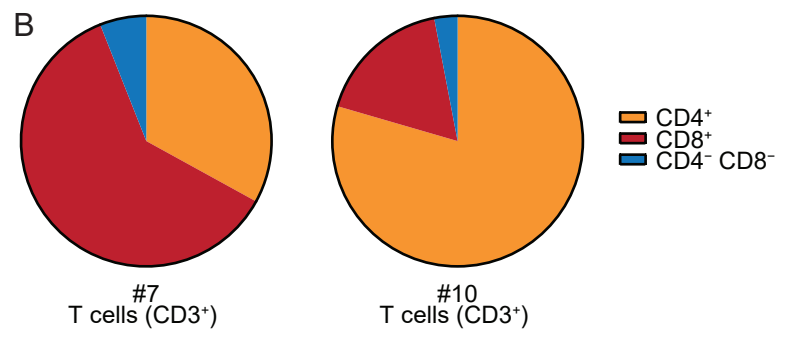

Supplement: Supplementary file 1 [file cells-14-00632-s001.zip › cells-3569171-supplementary/Supplemental Files/Supplemental Figures.pdf]
